# Global analysis of the genomic diversity, antimicrobial resistance and potential vaccine candidates carried by the major global bovine pathogen Streptococcus uberis

**DOI:** 10.1099/mgen.0.001441

**Published:** 2025-07-25

**Authors:** Adrianna M. Turner, Lucy Li, George Taiaroa, Ian R. Monk, Peter Mansell, Benjamin P. Howden, Claire L. Gorrie, Glen P. Carter

**Affiliations:** 1 Department of Microbiology and Immunology, The University of Melbourne at the Peter Doherty Institute for Infection and Immunity, Melbourne, VIC, Australia; 2 Department of Infectious Diseases, The University of Melbourne at the Peter Doherty Institute for Infection and Immunity, Melbourne, VIC, Australia; 3 Faculty of Science, Melbourne Veterinary School, The University of Melbourne, Melbourne, VIC, Australia; 4 Microbiological Diagnostic Unit Public Health Laboratory, Department of Microbiology and Immunology, The University of Melbourne at the Peter Doherty Institute for Infection and Immunity, Melbourne, VIC, Australia; 5 Centre for Pathogen Genomics, The University of Melbourne, Melbourne, VIC, Australia

**Keywords:** population genomics, *Streptococcus uberis*, vaccine

## Abstract

*Streptococcus uberis* is a leading cause of bovine mastitis, resulting in large economic losses and welfare issues for affected cows. However, relatively little is known about the global distribution and emergence of bovine mastitis-causing lineages and dissemination of antimicrobial resistance or virulence genes in the species. Here, we present a global framework for *S. uberis* based on whole-genome sequencing data from isolates across four continents (*n*=1,070). Internationally, *S. uberis* isolates show extensive genetic heterogeneity driven by homologous recombination and high levels of accessory genome content. Within the population, 35% (*n*=374/1,070) of isolates had at least one acquired antimicrobial resistance gene, while 59% (*n*=631/1,070) of isolates harboured a mutation in the penicillin-binding proteins (*pbp2b* or *pbp2x*) associated with decreased penicillin susceptibility, the front-line antimicrobial for mastitis infections. All described *S. uberis* virulence factors and currently proposed vaccine candidates were investigated for carriage and sequence heterogeneity to guide potential vaccine development, with two vaccine candidates (GapC and Sua) and seven putative virulence proteins (Fba, FbpS, Vru, GlnA, SUB0888, SUB0241 and haemolysin-like protein) having low naturally occurring sequence variation and high (>99%) coverage. This study will facilitate improved genomic surveillance for the emergence of antimicrobial resistance and virulence in this important mastitis pathogen.

**Impact Statement**

Despite being a leading cause of bovine mastitis, little is known about the *Streptococcus uberis* population structure and distribution of antimicrobial resistance and virulence genes. Within this highly diverse collection of global *S. uberis*, this study identifies that almost 60% of isolates harbour a resistance-associated mutation to the front-line treatment, penicillin, and 35% of isolates harbour at least one antimicrobial resistance gene. Although there are ongoing efforts to develop an effective *S. uberis* vaccine for bovine mastitis, no studies have analysed the diversity of these virulence genes in a global population. In this study, we identify two proposed vaccine candidates and seven putative virulence genes that are genetically conserved in *S. uberis* and could serve as targets for future research. Here, we present a genomic framework that can be used for future surveillance of this global mastitis pathogen.

## Data Summary

Genome sequences are deposited in GenBank under BioProject PRJNA1173866, and the accession numbers and sample data are available in Table S1 (available in the online Supplementary Material). All supporting data, code and protocols have been provided within the article or through supplementary data files.

## Introduction

Bovine mastitis is the most common disease in dairy industries causing high economic losses due to the cost of treatment, decreased production of milk and culling of affected animals (estimated at US$35 billion) [1]. Since the introduction of the mastitis five-point plan in the UK during the 1970s, there has been a reduction in the prevalence of cow-associated pathogens (e.g. *Staphylococcus aureus* or *Streptococcus agalactiae*) that are primarily transmitted during the milking procedure [2]. However, this plan resulted in a shift in the aetiology of clinical mastitis isolates, with environmental pathogens increasing in prevalence since the source of infections is primarily related to the habitat (e.g. bedding material or pasture), rather than driven through cow-associated transmission. Globally, *Streptococcus uberis* is one of the most frequently isolated and difficult-to-handle environmental pathogens that cause acute clinical and recurrent bovine mastitis [2,3]. *S. uberis* is present in pastures and indoor environments, but contagious transmission has been observed through longitudinal studies via the mucosae of the digestive tract and faeces [4,5]. Bacteriological cure rates following therapy for clinical infections caused by *S. uberis* are reported to range between 64 and 91%, with factors such as animal condition and antimicrobial resistance influencing this [6]. While the majority of *S. uberis* remain susceptible to front-line *β*-lactam therapeutics, resistance to these has been reported in France [7], Germany [8] and New Zealand [9], suggesting that this resistance could disseminate globally.

Previous epidemiological studies investigating the genetic diversity of *S. uberis* using typing methods such as multi-locus sequence typing (MLST) or PFGE suggested that the population is highly diverse since almost all samples from a single herd can be a different sequence type (ST) [10,12]. Similar to other *Streptococcus* species [13], *S. uberis* is highly recombinogenic, with homologous recombination identified at six of the seven MLST alleles, rendering the scheme likely ineffective at inferring genetic similarity [14]. Recently, a new core genome MLST (cgMLST) scheme has been proposed for *S. uberis* that could provide an alternative to MLST-based typing [15]. Comparative genomics using whole-genome sequencing (WGS) has suggested a large degree of genomic plasticity in strains from either a single herd or geographic location [11]. However, since few studies have compared the broader *S. uberis* population, relatively little is known about the global distribution or emergence of bovine mastitis-causing lineages and dissemination of antimicrobial resistance and virulence-associated genes.

Preliminary within- and between-herd analyses indicate that almost all *S. uberis* isolates possess virulence factors promoting invasion of host tissue, evasion of the host immune response and internalization within the mammary gland [3,10, 11]. *S. uberis* can produce biofilms and a hyaluronic acid (HA) capsule, which are hypothesized to assist in invading mammary gland cells and result in chronic infection and cow-associated transmission [1,16,20]. These characteristics make *S. uberis* mastitis difficult to control, leading to the development of vaccination-based strategies to improve animal welfare and the economics of milk production [21,24]. In the UK, the inactivated *S. uberis* UBAC (HIPRA) vaccine is currently licensed for mastitis, but the results from the randomized control trials have not been reported. The successful development of an effective vaccine against *S. uberis* has been hampered by an incomplete understanding of host-pathogen dynamics and global genetic diversity, with no studies analysing the prevalence and variability of these vaccine candidates. With this aim, we analysed the genomes of all available * S. uberis* isolates to understand the diversity of the *S. uberis* population, the distribution of antimicrobial resistance and virulence genes and the evolution of proposed vaccine candidates.

## Methods

### Bacterial isolates

A global analysis included the generation of Australian *S. uberis* genomic data and integration of this with all available public data for the species. Sixty-three *S. uberis* isolates were randomly selected from a larger culture collection consisting of strains previously isolated from cows at seven dairy farms in the Gippsland and Western Melbourne areas of Victoria, Australia. No information on the clinical status of the cows or somatic cell counts associated with each isolate was available. For publicly available isolates, our aim was to capture the diversity of *S. uberis* circulating globally by including all published isolates as of March 2024. To be included, isolates needed to have short-read data available, geographic location and source (bovine/ovine or environmental). All reads, including Australian and international, were only included if they had a (1) sequencing depth of >50, (2) GC content of 37%±2% and (3) Phred quality (% >Q30) above 80 and confirmed to be *S. uberis* with the Kraken2 database (v2.1.2) [25] (Table S1).

### DNA extraction and WGS

Genomic DNA was extracted from a single colony using a JANUS automated workstation (PerkinElmer) and Chemagic magnetic bead technology (PerkinElmer). Genomic DNA libraries were prepared using the Nextera XT kit according to the manufacturer’s instructions (Illumina, San Diego, CA, USA). WGS was performed using the Illumina NextSeq platform, generating 150 bp paired-end reads. All sequencing data are available on the NCBI Sequence Read Archive PRJNA1173866.

### Phylogenetic analysis

The 1,070 Australian and international *S. uberis* were mapped to the reference genome NZ01 isolated from a cow with a clinical case of bovine mastitis in New Zealand (NCBI accession: CP022435) [26] using snippy (https://github.com/tseemann/snippy) (v4.4.5), applying a minfrac value of 10 and mincov value of 0.9. This reference was selected as it was a publicly available complete genome collected locally and is part of the largest *S. uberis* phylogroup (bp 2). A maximum-likelihood phylogenetic tree was inferred using IQ-TREE (v2.1.4) [27] with a general time-reversible (GTR+G4) substitution model, including invariable sites as a constant pattern and 1,000 bootstrap replicates. Recombination masking was not performed for the *S. uberis* species maximum-likelihood tree due to the small size of the resulting core alignment. The species alignment contained a core alignment length of 1,863,842 bp and included 79,751 variant sites. For each phylogroup, the core alignment used a within ‘cluster reference’ (complete genome of the same cluster) to maximize core-SNP alignment length (Table S2), and recombination was masked from the alignment using Gubbins (v3.3.1) [28]. Recombination versus *r/m* ratios for each lineage were calculated as the average *r/m* including all isolates within the phylogroup. For the species, *r/m* was determined by averaging across all major phylogroups. All trees were mid-point rooted and visualized in R (v4.3.0, https://www.r-project.org/) using phangorn [29] (v2.5.5), ape [30] (v5.4), ggtree [31] (v2.3.4) and ggplot (v3.3.2).

### *De novo* assemblies and phylogenomic clustering

*De novo* assemblies were constructed using SPAdes [32] (v3.13.0) with default settings. *In silico* MLST was assigned using the program mlst (https://github.com/tseemann/mlst) (v2.19.0). Evolutionary related clusters were performed using fastbaps (v1.0.8) [33] from the core alignment conditioned on the population phylogenetic tree (from above), with these designations used in downstream analyses.

### Pangenome identification and comparison with *Streptococcus pyogenes* pangenome

All assemblies were annotated using the run_prokka function in Panaroo [34] (v1.2.10) with clean mode set to strict, which annotates each sample with the same gene model using Prokka [35] (v1.14.6). The *S. uberis* pangenome was defined using Panaroo (v1.2.10), which utilizes a pangenome graph-based approach for pangenome clustering. Core genes were defined as genes present in ≥99% of genomes, shell accessory genes between 15% and 99% of genomes and cloud accessory genes in <15% of genomes. Clusters of Orthologous Groups (COG) functional categories were assigned to core genes using eggNOG-mapper (v2.1.2) with default Diamond mode. COG categories J, K, L, A, B and Y were summarized as genes involved in ‘information storage and processing’, categories T, D, V, U, M, N, O, W and Z were summarized as genes involved in ‘cellular processing and signalling’ and categories C, G, E, F, H, I, P and Q were summarized as genes involved in ‘metabolism’.

A merged pangenome with *S. pyogenes* was then used to map functional classes of core genes across species. The *S. pyogenes* pangenome was previously defined [13,36], with the same methodology used for *S. uberis*. Panaroo [34] (v1.2.10) was used to merge the pangenome graphs for each species. Metabolic differences between *S. uberis* and *S. pyogenes* were inferred by searching for well-defined complete KEGG modules present in one species but not the other using eggNOG-mapper (v2.1.2) [37].

### Antimicrobial resistance and virulence gene screening

The genome assemblies for all isolates were screened for acquired antimicrobial resistance determinants using abriTAMR [38] (v1.0.18) with default settings. Mutations in the penicillin-binding proteins were identified using snippy (as above). The presence of virulence genes was determined by blastn (v2.12.0) on all genome assemblies, based on a 70% nt cut-off over 70% of the gene length. Allelic variation was examined by plotting tblastn scores relevant to the query reference sequence (0140J), the first reference *S. uberis* genome. The molecular signatures of selective constraints for the putative virulence genes were investigated using the d*N*/d*S* ratio in HyPhy (v2.5.58) [36].

### Data visualization

All figures were generated in R (v4.3.0) (https://www.r-project.org/) [39] using packages listed in Methods or the tidyverse suite (v2.0.0) [40].

## Results

### Global *S. uberis* population structure

**Fig. 1.**
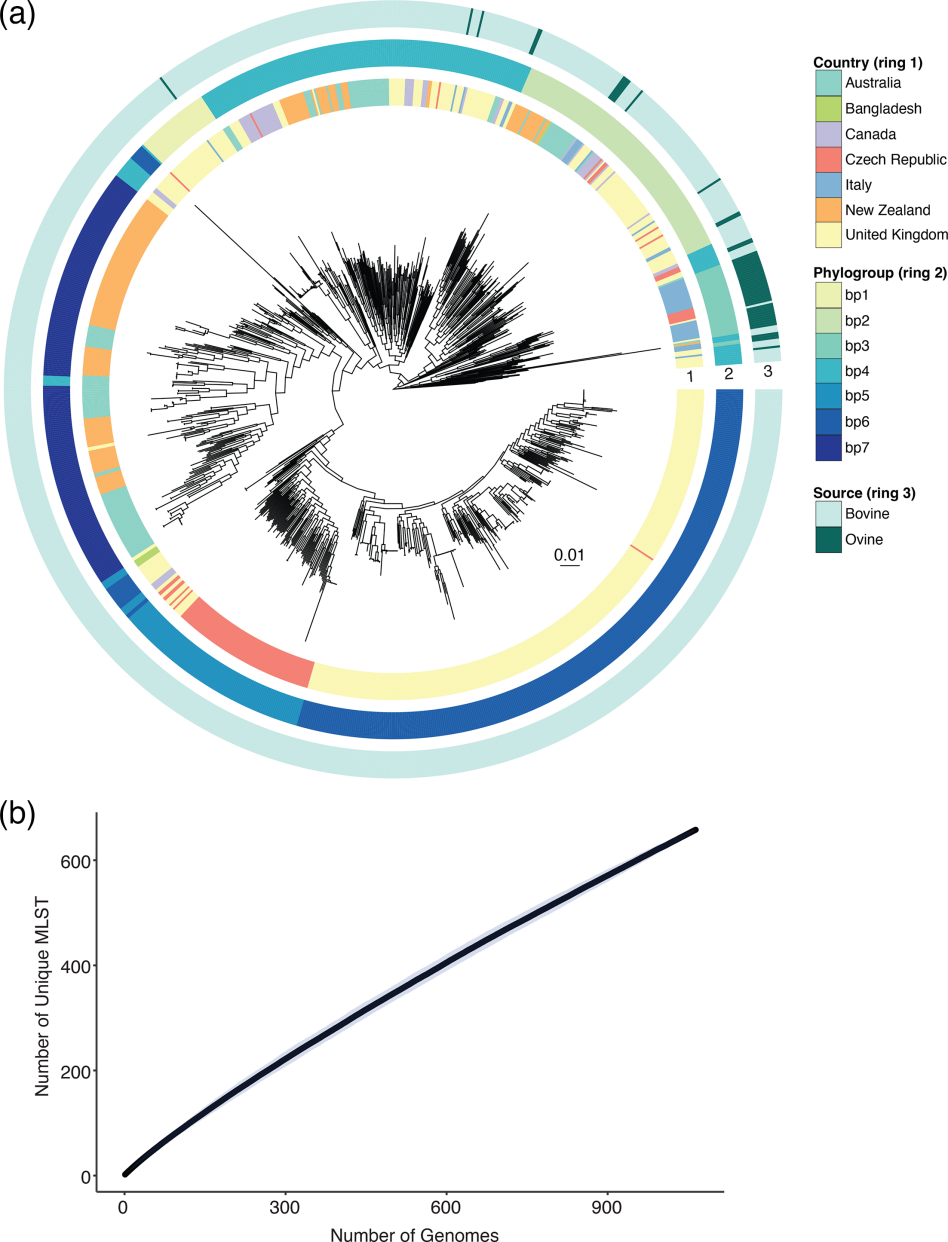
(a) Maximum-likelihood core SNP-based phylogeny of *S. uberis* (*n*=1,070) inferred from 79,751 SNPs. Overlaid are the (1) country of isolation, (2) results of Bayesian analysis of population structure clustering and (3) host. The scale bar indicates the number of nt substitutions per site. (**b)** MLST accumulation curve.

To understand the phylogenetic diversity within *S. uberis*, we performed WGS on strains isolated in Australia (*n*=68) and collated a diverse geographical and clinical (sub-clinical or clinical mastitis) database of published genome sequences (*n*=1,002). This collection consisted of 1,070 *S*. *uberis* isolates across 4 continents and 7 countries from both bovine (*n*=1,018) and ovine (*n*=52) origin (Table S1). The relationship between international *S. uberis* strains was investigated with a maximum-likelihood phylogeny inferred from an alignment of 79,751 core genome SNPs (Fig. 1a). This revealed a deep branching star-like population structure, suggesting that *S. uberis* is split into many distinct lineages. Indeed, *in silico* MLST identified more than 250 STs within the 1,070 isolates, with the majority of identified STs harbouring a single isolate (*n*=154) or a novel ST (*n*=405). Plotting an accumulation curve suggests that further MLSTs will continue to be detected as more *S. uberis* are isolated (Fig. 1b). Recombination was detected at all seven MLST alleles (*arcC*, *ddl*, *gki*, *recP*, *tdk*, *tpi* and *ydiL*), indicating that MLST alone should not be used in * S. uberis* genetic comparisons or clustering, such as with transmission or outbreaks. Indeed, the largest clusters of isolates within the same ST (ST6, ST60, ST63, ST307, ST360 and ST741) were polyphyletic (Fig. S1).

Applying a hierarchical Bayesian analysis of population structure, we identified seven distinct clusters of evolutionary related lineages, herein termed phylogroups, that were concordant with the inferred phylogeny (Fig. 1a). Overlaying the geographical origin of the isolates indicated that every phylogroup consisted of *S. uberis* isolates from multiple locations, suggestive of a highly diverse global population (Fig. S2). When overlaying the host of *S. uberis* infection, there was no clear association with bovine or ovine; however, bp3 consisted of isolates from only ovine origin (Fig. 1a). An analysis of the genome-wide relationships within this global dataset identified 1,433 core genes present in ≥99% of genomes and an ‘open’ pangenome of 8,512 unique protein-coding sequences. This pangenome estimate is considerably larger than previous *S. uberis* reports [10,11], highlighting how further genes will continue to be detected as additional genomes are sequenced. The majority of accessory genes were rare, with 76.1% of genes (*n*=6,478/8,512) found in ≤15% of *S. uberis* and 15% (*n*=1,284/8,512) found in only one genome.

### The *S. uberis* pangenome differs from the human-adapted *S. pyogenes* pangenome

**Fig. 2.**
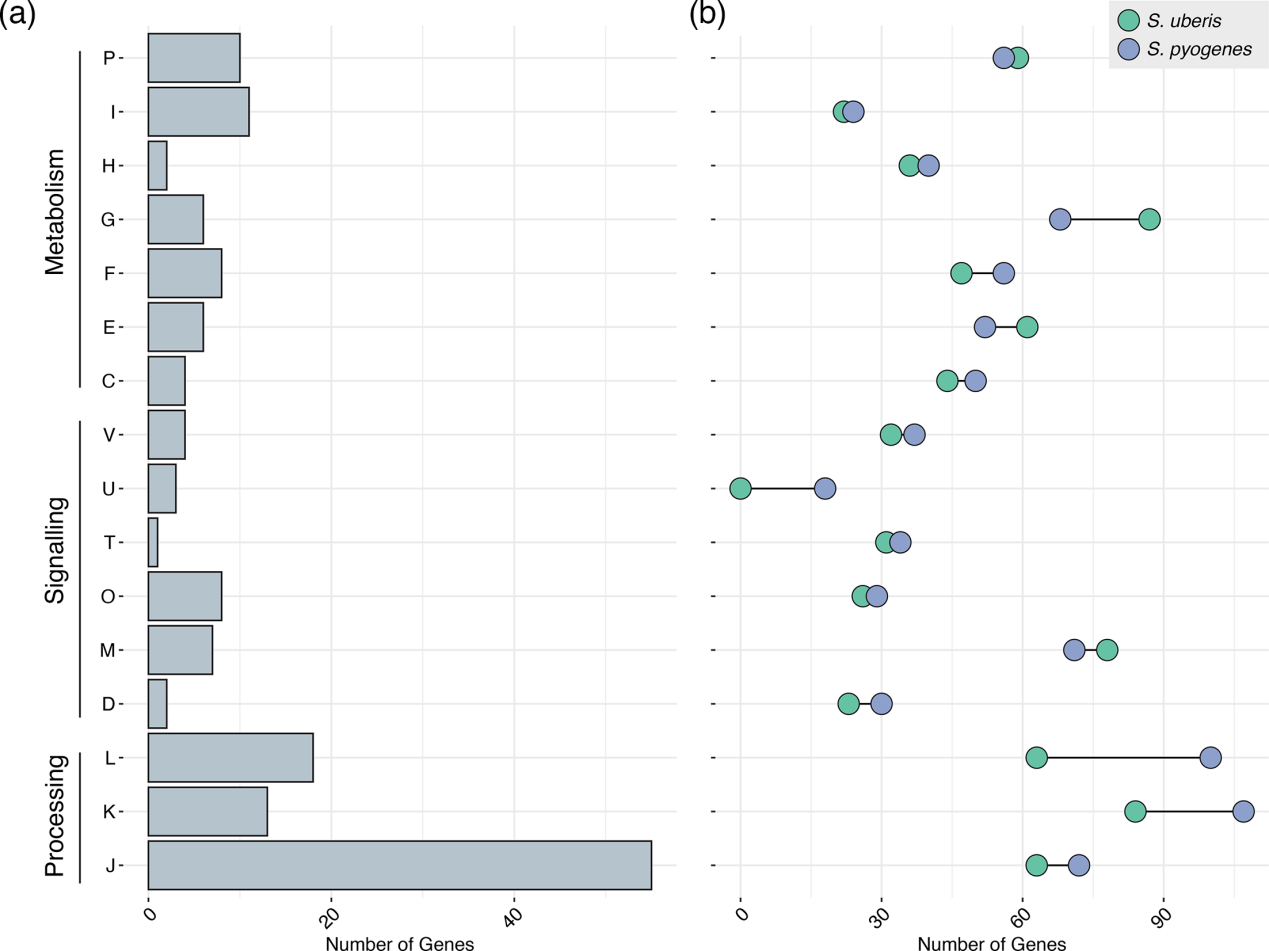
**(a)** Plot of the shared *S. uberis* and *S. pyogenes* pangenome based on predicted COG category. (**b)** Differences in the specific gene content in *S. uberis* (green) and *S. pyogenes* (blue) for each COG category.

We then compared the *S. uberis* pangenome to the genetically related, but human-adapted, *S. pyogenes* pangenome to identify signatures of adaptation present in *S. uberis*. While *S. pyogenes* has been shown to share a large core genome with other human-adapted streptococci (such as *Streptococcus dysgalactiae* subsp. *equisimilis* with 1,166 core genes [36], only 329 genes were shared in a merged pangenome between *S. uberis* and *S. pyogenes* (see Methods). This compromised 19.8% of the *S. uberis* core genome and 18.8% of the *S. pyogenes* core genome, suggesting large differences in gene content between the two streptococcal species. Examining the function of differing KEGG pathways suggested that the majority were involved in ‘information storage and processing’ (group K and L) and ‘metabolism’ (group G) (Fig. 2a, b). Within the ‘information storage and processing’ category, the largest differences were in the presence of different transcriptional regulators (*n*=17 in *S. pyogenes* and *n*=11 in *S. uberis*) and clustered regularly interspaced short palindromic repeats (CRISPR) systems, with 49% of *S. pyogenes* isolates harbouring a CRISPR-Cas2 cluster and 59% of *S. uberis* isolates harbouring a CRISPR-Cas1 (26%) or CRISPR-Cas2 cluster (33%). Within the ‘metabolism’ category, the predicted metabolic differences suggested that only 16.6% of *S. uberis* and 15.1% of *S. pyogenes* metabolic genes were genetically similar. The *S. uberis* pangenome contained additional phosphoenolpyruvate-dependent sugar phosphotransferase systems for binding galactose (*lacA*, *lacB* and *lacR*) and lactose (*licA*, *licB* and *licC*), which are abundant in bovine milk and were absent in *S. pyogenes. S. uberis* also harboured more genes in the glycoside hydrolase family 1 than * S. pyogenes* (*n*=10 versus *n*=4), suggesting that *S. uberis* has the potential to hydrolase a larger range of sugars, potentially related to survival in both host and environmental niches.

### Recombination is a significant driver of *S. uberis* evolution

Several studies [10,14] have suggested that *S. uberis* is highly recombinogenic. Therefore, to assess the relative contribution of homologous recombination on individual lineages, we quantified the genome-wide rate and fragment length of recombination within the seven phylogroups (Figs S3 and S4). The microevolution for each lineage was assessed by mapping to a phylogroup-specific reference genome. The average number of SNPs observed within the 6 phylogroups was 1,876 (range: 1–8,705), of which an average of 40.7% of SNPs (range: 31.2–48.8%) were found to be vertically inherited within a phylogroup (Table S2, Fig. S3A–C). Overall, the mean ratio of recombination-derived mutation versus vertically derived mutation (*r/m*) was found to be 4.6 (median: 3.7), which is significantly greater than 1 (one-sample Wilcoxon test, *P*<0.05), suggesting that the primary driver of variation in *S. uberis* is through recombination. The average number of recombination events per phylogroup was 38 (range: 0–112) (Table S2). Large recombination events (>5,000 bp) were infrequent, with the majority of recombination fragments short in length (Fig. S4A, B). The average length of recombination fragments in each of the phylogroups was 2,638 bp, ranging from 2 to 90,237 bp. Collectively, these data highlight that evolution in the *S. uberis* population is primarily driven by homologous recombination events and high heterogeneity in accessory genome content.

### Distribution of genomic determinants of antimicrobial resistance

**Fig. 3.**
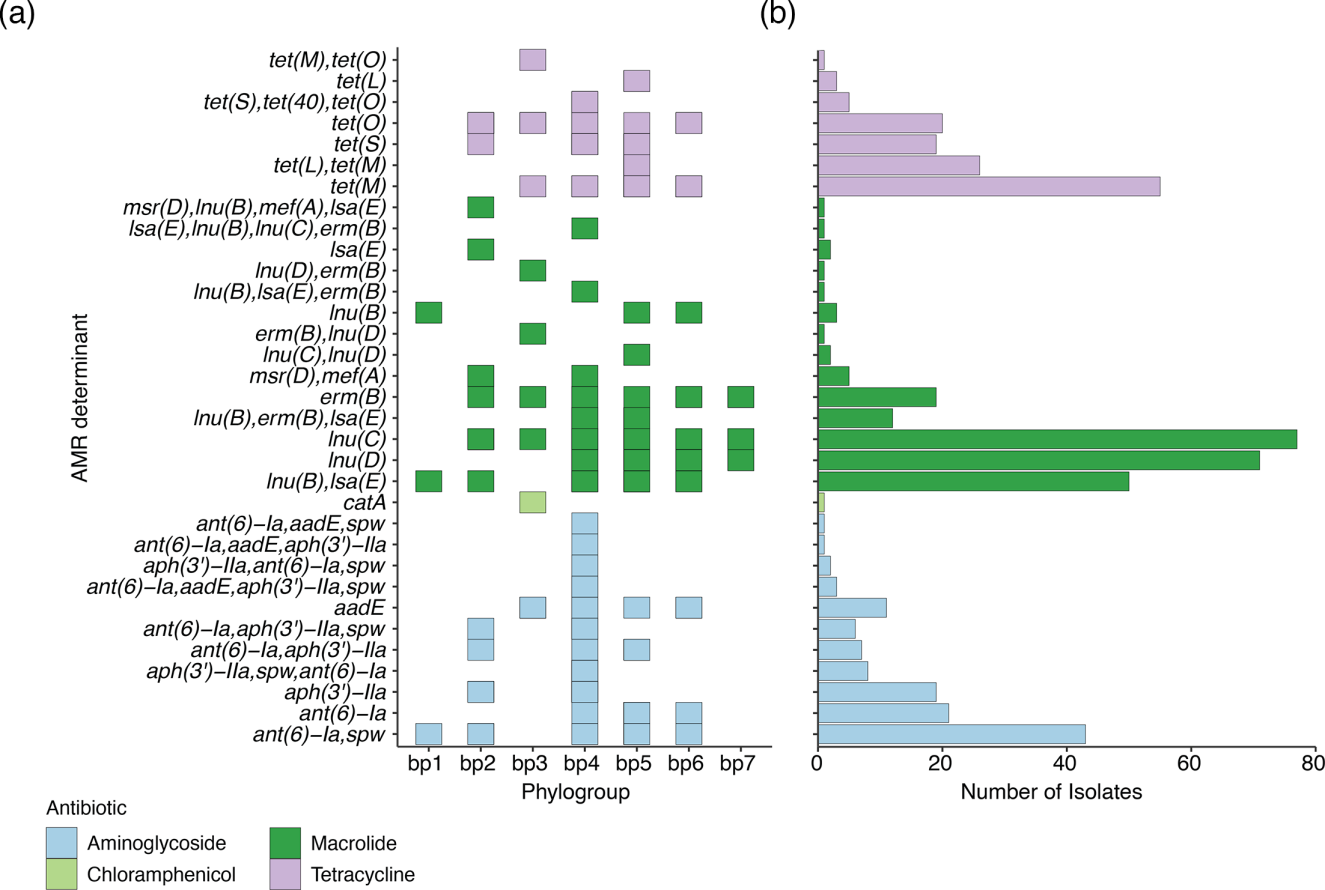
(a) Heatmap of combined overall genotypic AMR profiles observed for each *S. uberis* strain. This represents the broad AMR profile for each phylogroup. The AMR genes are coloured according to their antimicrobial family. (**b)** Bar plot showing the frequency of each AMR determinant according to each antimicrobial family. AMR genes that were co-occurring in the same class are grouped together, i.e. *tetM* and *tetO*.

The presence of acquired antimicrobial resistance (AMR) genes has been reported in previous *S. uberis* studies; however, the distribution of these determinants within the population or association with particular lineages is incompletely defined [6,10, 41]. When we genetically assessed our collection of *S. uberis*, 380/1,070 (35.0%) isolates in all phylogroups contained at least 1 acquired AMR determinant. Amongst the 380 isolates, 34 different genomic AMR determinants were identified (Fig. 3a, b). This consisted of genes predicted to confer resistance to aminoglycosides, chloramphenicol, macrolides and tetracyclines. The diversity of AMR genotypes was further reflected in 64 different combinations of AMR determinants within the 1,070 *S*. *uberis* isolates. The most common AMR genes were *ant*(6)-la and *aph*(3′)-lla (*n*=79 and *n*=29, respectively; aminoglycoside), *tet* [*tetL* (*n*=29), *tetM* (*n*=55) and *tetS* (*n*=24); tetracycline] and *lnu* [*lnuC* (*n*=71) and *lnuD* (*n*=77); lincosamide]. No AMR determinants were specific to a certain phylogroup. However, phylogroup bp4 had significantly more (*P*<0.01; median 4) resistance genes than the other phylogroups (median: bp2=2, bp3=2, bp5=2, bp6=2 and b7=1), consisting of isolates from Australia, Czech Republic, Italy and New Zealand.

Individual studies in Canada [42], France [7] and New Zealand [9] have observed an increase in *β*-lactam resistance in *S. uberis*. Given the clinical importance of *β*-lactams for the treatment of mastitis, we analysed our *S. uberis* dataset for mutations in the penicillin-binding proteins *1a*, *1b*, *2a*, *2b* and *2x* that are associated with decreased penicillin susceptibility in streptococci. The diversity of *pbp* alleles was extensive, with 269 missense SNPs identified across the 5 *pbp* genes. Of the 5 genes, *pbp2b* was the most diverse, with 74 unique alleles identified, followed by *pbp2a* with 58 unique alleles, *pbp1b* with 55, *pbp1a* with 46 and, lastly, *pbp2x* with 37 unique alleles. The majority (>75%) of unique *pbp* alleles were shared between the different phylogroups. The *pbp2* mutations [*pbp2b* N366I (*n*=203) and T402I (*n*=184) and *pbp2x* E381K (*n*=535), Q554E (*n*=527), V590A (*n*=458) and G600E (*n*=531)] associated with decreased penicillin susceptibility were distributed throughout the phylogroups, with isolates typically harbouring both *pbp2b* mutations or all four *pbp2x* mutations, suggesting that these mutations co-evolve or are gained through recombination together (Fig. S5).

### The HA capsule is variably present within the global *S. uberis* population

**Fig. 4.**
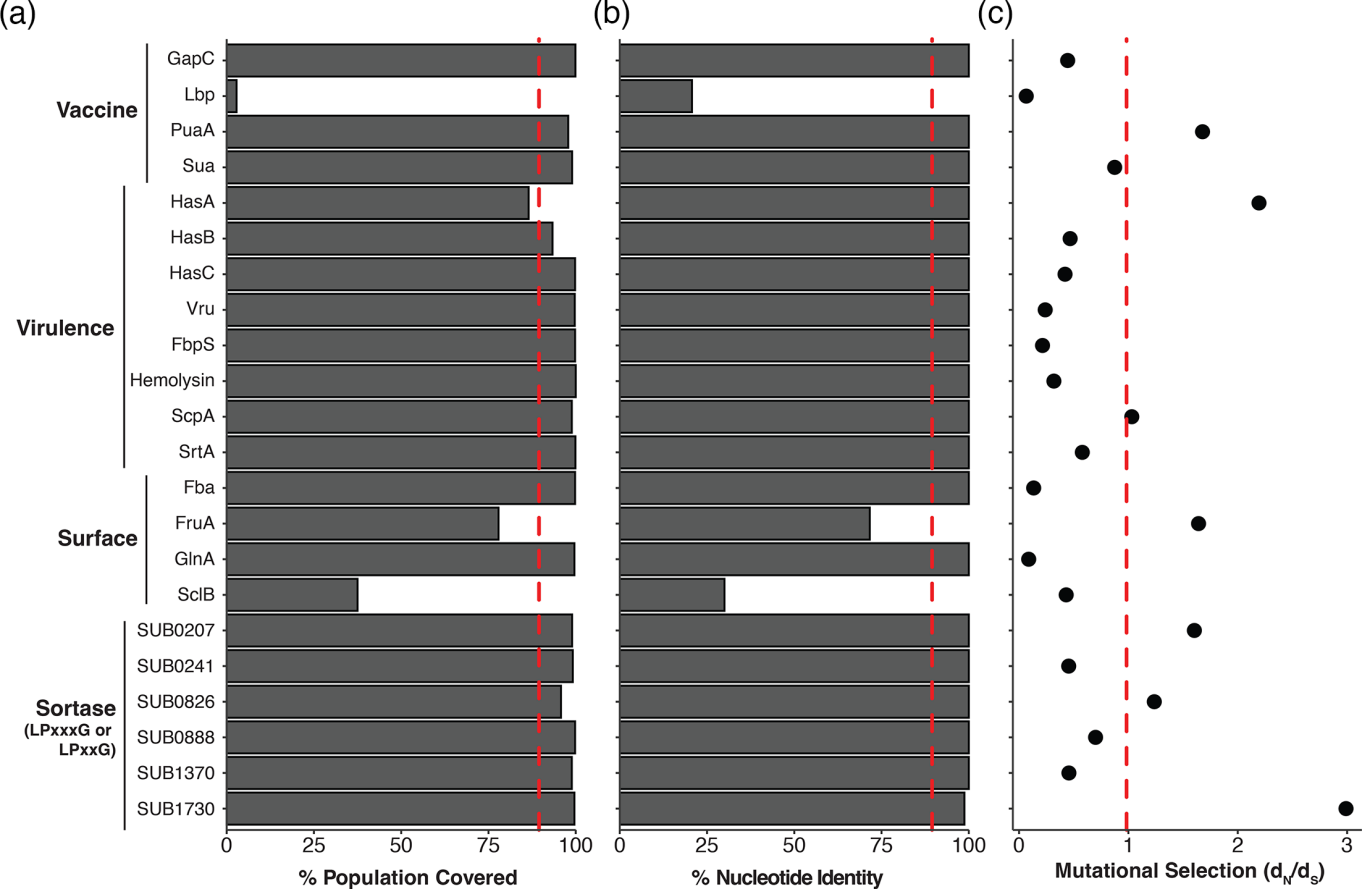
(a) The putative virulence genes were screened in our *S. uberis* (*n*=1,070) collection for the presence in the population (based on a 70% nt length of the 0140J reference sequence) and (**b)** nt identity (based on a 70% nt identity of the 0140J reference sequence). The red dashed lines represent the 90% coverage and nt identity thresholds, respectively. (**c)** The d*N*/d*S* ratios (dots) for the putative virulence genes. The red dashed line represents the cut-off for purifying selection (a ratio of 1).

The HA capsule plays a significant role in the pathogenesis of Group A *Streptococcus* (GAS) [43], with variable amounts of HA capsule observed in *S. uberis* strains isolated from cases of bovine mastitis. While the HA capsule may not be required for clinical mastitis [44], it is hypothesized that the capsule may prevent desiccation in the environment, thereby increasing the chance for colonization or infection. The arrangement of the HA biosynthetic pathway in *S. uberis* differs from other GAS with the ‘operon’ consisting of the hyaluronan synthase *hasA* (SUB1697) and uridine diphosphate (UDP)-glucose dehydrogenase *hasB* (SUB1696), with the UDP-glucose pryphosphorylase *hasC* (SUB1692) separated from *hasAB* in the genome by ~3 kb of sequence. Similar to other GAS, only *hasA* is required for the production of the HA polymer from UDP-glucuronic acid and *N*-acetylglucosamine sugar precursors [16]. Previous genetic and phenotypic studies in *S. uberis* have suggested that the *hasABC* genes are part of the * S. uberis* core genome, and while varying amounts of HA capsule are produced, this may be related to mutations or environmental factors. However, we observed that both *hasA* and *hasB* were absent in phylogroup bp2 and bp3, similar to that observed in a previous *S. uberis* analysis (*hasA* present in 24/27 isolates) [10]. All isolates harboured the *hasC* gene (SUB1692), as well as paralogues of *hasB* (SUB1027; *hasB2*) with an unknown function. Despite the variability in the presence of the *hasA* and *hasB* genes, isolates that carried the operon harboured a similar genetic context (Fig. S6). The *hasA* and *hasC* genes were highly conserved at the nt level [98.8–100% based on SUB1697 (*hasA*) and SUB1692 (*hasC*) reference sequences], but the *hasB* gene was much more variable [50.25–100% based on SUB1696 (*hasB*)]. The potential virulence regulatory gene *vru* (SUB0144), a homologue of *mga* (GAS) which regulates *hasA* and *hasB* expression in *S. uberis* [45], was present in all isolates with high nt identity (>95% average) (Fig. 4a). A previously identified deletion in the promoter region of *vru* (position −76 to −79; TAATA to T) hypothesized to be associated with sub-clinical mastitis [11] was identified in 392 strains (36.1%, *n*=392/1,070), with the deletion leading to asymptomatic carriage in GAS. Overall, these results suggest that the HA capsule in *S. uberis* is not part of the core genome and is instead associated with certain phylogenetic lineages.

### Distribution and evolution of *S. uberis* vaccine candidates

Little is known about the conservation of proposed vaccine antigens, despite previous efforts to develop an effective vaccine for preventing *S. uberis* mastitis infections [21,24]. To examine the natural variation of proposed *S. uberis* vaccine antigens and putative virulence factors within this diverse dataset, gene carriage (presence or absence) and sequence variation of all *S. uberis* vaccine targets and virulence genes were determined.

We firstly assessed the conservation of the previously proposed vaccine antigens for *S. uberis* [21,24]. This included glyceraldehyde-3-phosphate dehydrogenase (SUB1630; *gapC*), lactoferrin-binding protein *lbp* (SUB0145), plasminogen activator *pauA* (SUB1785) and adhesion molecule *sua* (SUB1635) (Fig. 4a, b) [23,46, 47]. While *gapC* and *sua* were present in the core genome (>99% of strains), *pauA* and *lbp* were present at more varying frequencies, at 97.8% (*n*=1,044/1,070) and 4.3% (*n*=46/1,070) of isolates, respectively. In addition to being ubiquitous within the *S. uberis* population, an ideal vaccine candidate would exhibit low levels of naturally occurring sequence variation within a genetically diverse dataset. Only *gapC*, *pauA* and *sua* exhibited high levels (>90%) of sequence (nt level) conservation. Using a ratio of non-synonymous to synonymous mutations (d*N*/d*S*) to infer positive or negative selection, *puaA* had signatures of positive selection (d*N*/d*S*=1.67), while the lactoferrin-binding protein *lbp* had almost no mutational selection (d*N*/d*S*=0.02), due to the very low prevalence of this gene in the *S. uberis* population (Fig. 4c).

We then expanded our analysis to include described *S. uberis* virulence factors and surface proteins. Several putative virulence genes were present in the *S. uberis* population core genome, such as the fibronectin-binding proteins (SUB0330; *fbA* and SUB1111; *fbpS*), glutamate metabolism protein (SUB1626; *glnA*), haemolysin-like protein (SUB1273), C5a peptidase (SUB1154; *scpA*) and sortase *srtA* (SUB0881) (Fig. 4a). However, two virulence factors previously suggested to be conserved in *S. uberis* were present at more varying frequencies: fructonase *fruA* (*n*=831 or 77.8%; SUB0135) and surface protein *sclB* (*n*=401 or 37.5%; SUB1095) [21]. To expand our dataset of potential vaccine candidates, we searched for new sortase-anchored proteins (motif LPxxG or LPxxxD) and identified six genes (SUB0207, SUB0241, SUB0826, SUB0888, SUB1370 and SUB1730, respectively) present in ≥95% of strains and genetically conserved (>90%).

Overall, 8 of the 18 putative virulence factors or surface-exposed proteins were carried by >99% of the 1,070 strains and exhibited low levels of allelic variation (<2% sequence divergence) (*fbA*, *fbpS*, SUB0888, *vru*, *glnA*, SUB0241, SUB1730 and haemolysin-like protein) (Fig. 4a). Of these eight core genes, seven exhibited signatures of purifying selection (i.e. reducing genetic diversity) with d*N*/d*S* ratios of 0.13 (*fbA*), 0.21 (*fbpS*), 0.69 (SUB0888), 0.24 (*vru*), 0.08 (*glnA*), 0.45 (SUB0241) and 0.32 (haemolysin-like protein) (Fig. 4b). These findings suggest that the proteins encoded by these seven genes should be investigated further as potential vaccine candidates, given their high degree of genetic conservation in a highly diverse species.

## Discussion

Here, we provide a genomic framework for which a deeper understanding of the *S. uberis* population can be developed for more informed genomic tracking and surveillance of virulence and AMR in this important bovine pathogen. Currently, there are ongoing efforts to develop a safe and efficacious *S. uberis* vaccine for the prevention of bovine mastitis. As we demonstrate here, one of the hurdles to the development of an effective *S. uberis* vaccine suitable for worldwide use is the extensive genetic diversity of the global *S. uberis* population due to a large dispensable gene pool arising from recombination occurring across the genome. The inclusion of isolates from wider sources, such as both bovine, ovine and environmental, will be important to extend the understanding of the population further, so future studies can begin to separate clones and genetic factors associated with clinical mastitis, colonization or the environment. Recombination has previously been suggested to be prevalent in *S. uberis* through microarray analysis [14], but never analysed at a genome-wide population level. Our findings suggest a major role for homologous recombination of small-length DNA fragments (single or few genes) in driving the evolutionary dynamics of *S. uberis*, indicating that the evolution of new lineages is more likely to arise by recombination rather than by mutation. Understanding the impacts of recombination and, therefore gene exchange, in the *S. uberis* population has important consequences for the development of a future vaccine, where the emergence of vaccine escape clones has been documented in similarly recombinant species like *Streptococcus pneumoniae* [48].

Our analyses here suggest that the genes encoding putative virulence factors, as well as genes associated with antimicrobial resistance, are not uniformly distributed in the *S. uberis* population. In particular, the second largest lineage (bp4) representing isolates from all analysed regions consisted of strains with the highest number of virulence and antimicrobial resistance genes, suggestive of a phylogroup with potentially both increased virulence and antimicrobial resistance that warrants further surveillance and phenotypic investigation. Prior assumptions on the prevalence of different virulence factors, and the identification of potential vaccine antigens, have largely been based on the first reference *S. uberis* genome 0140J [3]. Our analyses indicate the 0140J reference strain forms part of the fourth largest lineage (bp5), most representative of bovine isolates from the European continent (Czech Republic and the UK). However, due to the large accessory genome of *S. uberis*, some of the previously described putative virulence factors in 0140J are variably present in the overall *S. uberis* population. Most notable was the lactoferrin-binding protein (*lbp*) that was used in a preliminary bovine challenge model [21] but is absent in 95% of *S. uberis* isolates in this study. Our analyses improve the understanding of potential vaccine antigen gene carriage and sequence variation within the global *S. uberis* population, with our data suggesting that seven, Fba, FbpS, Vru, GlnA and haemolysin-like protein, virulence factors and two surface-associated proteins, SUB0888 and SUB0241, are part of the *S. uberis* core genome and display a high degree of genetic conservation. Prior research has suggested that the fibronectin-binding protein (FbA) can elicit an immunological and protective response in a murine model [22]. Given the high level of conservation (99.5% nt identity) of this gene in the *S. uberis* population, further studies investigating FbA in bovine models are warranted. No studies have analysed the immunological response for the fibronectin-binding protein (FbpS), glutamate metabolism protein (GlnA), Vru regulator, haemolysin-like protein or the two-surface exposed proteins SUB0241 and SUB0888. Understanding the potential of these proteins as possible vaccine antigens is needed, as well as further investigations into the host-pathogen relationship to identify new virulence genes that are potentially present and conserved in the *S. uberis* population.

The global population structure of *S. uberis* is highly diverse, which is driven by recombination occurring across the entire genome. Current *S. uberis* studies use MLST for understanding epidemiology and outbreaks within and between herds. While MLST may be able to cluster isolates that are geographically related, our data here suggests that the scheme is not scalable at a population level. Further, isolates of the same MLST form independent clades in an SNP-based phylogeny, suggesting that the scheme is ineffective at genetic clustering. These findings are in agreement with a recent study that showed a high genetic diversity of *S. uberis* when analysed by a new cgMLST scheme where 1,012 isolates were clustered into 932 unique cgMLSTs [15]. Effective epidemiological surveillance of *S. uberis* necessitates the development of more sophisticated phylogenetic clustering methodologies, particularly those leveraging pan-genome analysis or split k-mer-based approaches.

This study provides a foundation and support for future work investigating ecological niche adaptation, pathogenicity and lineage diversification in *S. uberis* and will facilitate more deeply informed genomic surveillance for the emergence of virulence and AMR in this important mastitis pathogen.[Supplementary-material SM1 SM2]

## Supplementary material

10.1099/mgen.0.001441 Supplementary Material 1.

10.1099/mgen.0.001441 Supplementary Material 2.

## Abbreviations

cgMLST: core genome multi-locus sequencing typing
COG: Clusters of Orthologous Groups
CRISPR: clustered regularly interspaced short palindromic repeats
GAS: Group A *Streptococcus*
HA: hyaluronic acid
MLST: multi-locus sequence typing
ST: sequence type
WGS: whole-genome sequencing
