# Supplementary material for: Global analysis of the genomic diversity, antimicrobial resistance and potential vaccine candidates carried by the major global bovine pathogen Streptococcus uberis
Source: Microb Genom. 2025 Jul 25;11(7):001441. doi: 10.1099/mgen.0.001441 (PMC12452173; doi:10.1099/mgen.0.001441)
Supplement: Supplementary Material 2. [file mgen-11-01441-s002.pdf]

- Supplemental File -

Global analysis of the genomic diversity, antimicrobial resistance, and potential vaccine candidates carried by the major global bovine pathogen *Streptococcus uberis*

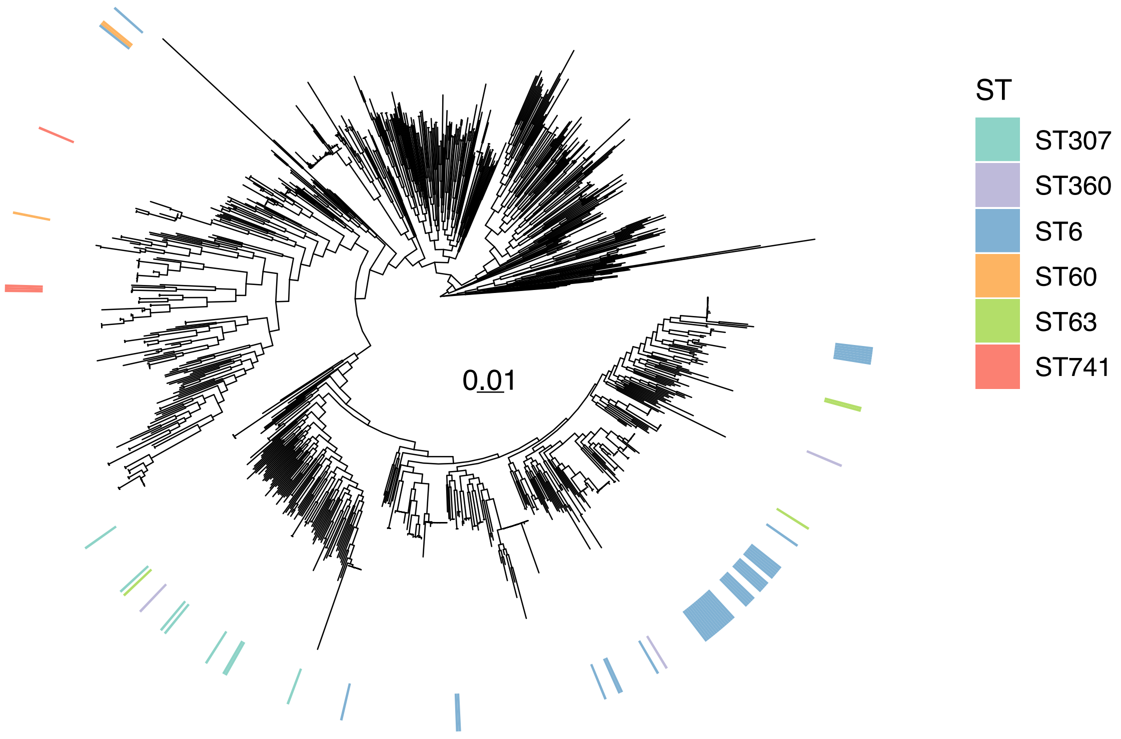

Supplementary Figure 1. Maximum-likelihood core-SNP-based phylogeny of *S. uberis* (n=1070) inferred from 79,751 SNPs, demonstrating the interspersing of sequence types (STs), rather than clonal clustering, throughout the tree.

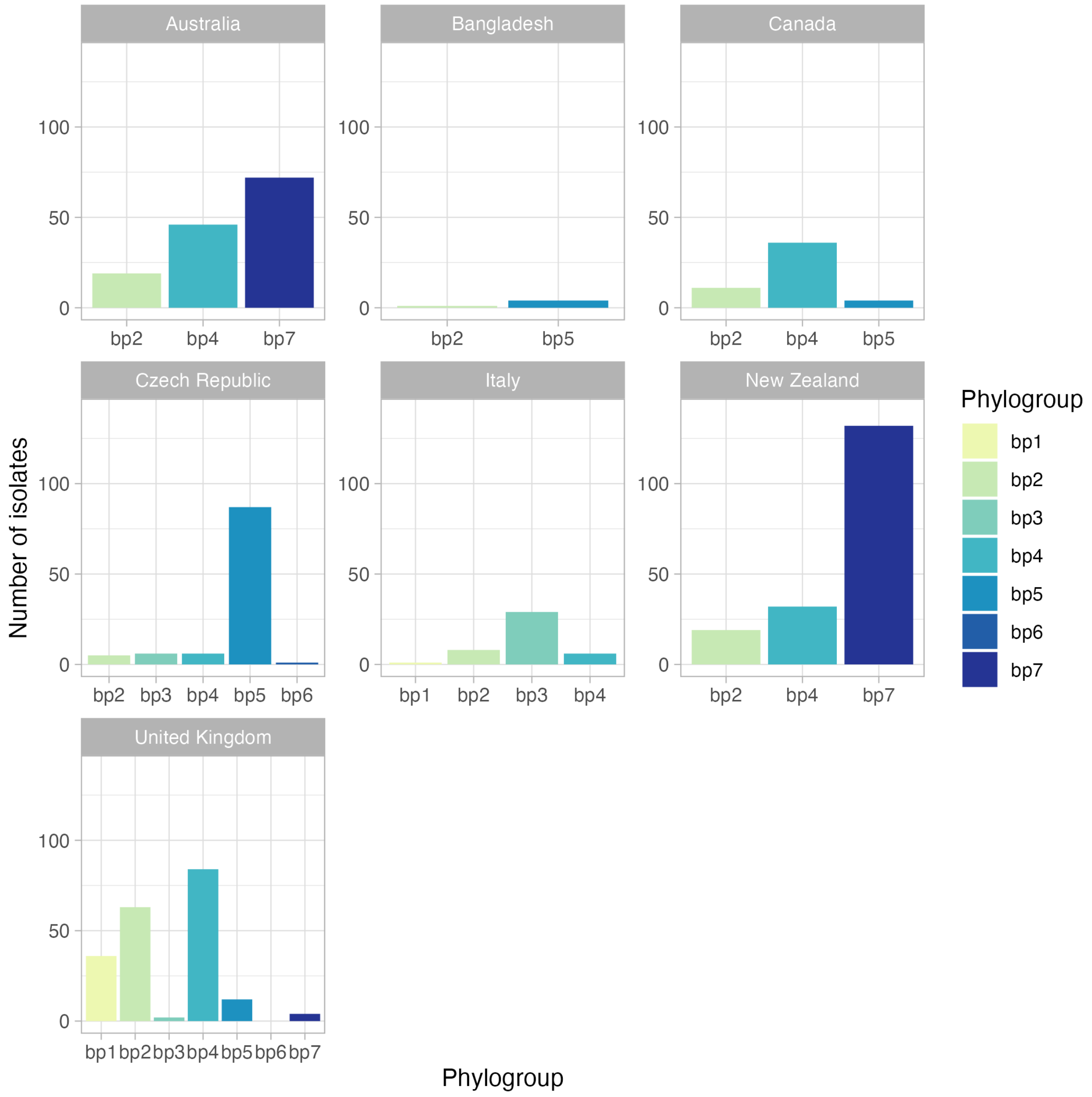

Supplementary Figure 2. Geographical sampling of the source of the 1070 genomes included in this study across 7 countries (4 continents). The phylogroup based on baps clustering (bp1-7) is on the x axis. The y axis for each country is on a different scale to aid in visualisation.

Supplementary Table 2. Frequency and relative rates of recombination within the phylogroups.

**Phylogroup**	**Recombination vs Mutation Calculations**					
**Baps designation**	**No. of isolates**	**Median No. of SNPs**	**Median Vertically inherited SNPs (range) (% total)**	**Median No. of recombination events**	**rho/theta ratio**	**r/m ratio**
**1**	44	110 (0-1,511)	129 (4-374) **(64%)**	34	0.03	5.4
**2**	126	848 (2-6,001)	260 (3-3,300) **(31.2%)**	35	0.03	8.4
**3**	37	52 (2-705)	168 (3-346) **(48.8%)**	38	0.11	2.1
**4**	210	449 (1-3,252)	850 (1-4,946) **(44.5%)**	36	0.12	3.7
**5**	107	643 (16-3,086)	754 (9-6,372) (**38.8%**)	30	0.16	6.4
**6**	339	1,051 (3-2,175)	660 (0-5,471) **(40.3%)**	49	0.09	2.5
**7**	208	493 (0-2,346)	259 (3-3,359) **(48.0%)**	32	0.12	3.5

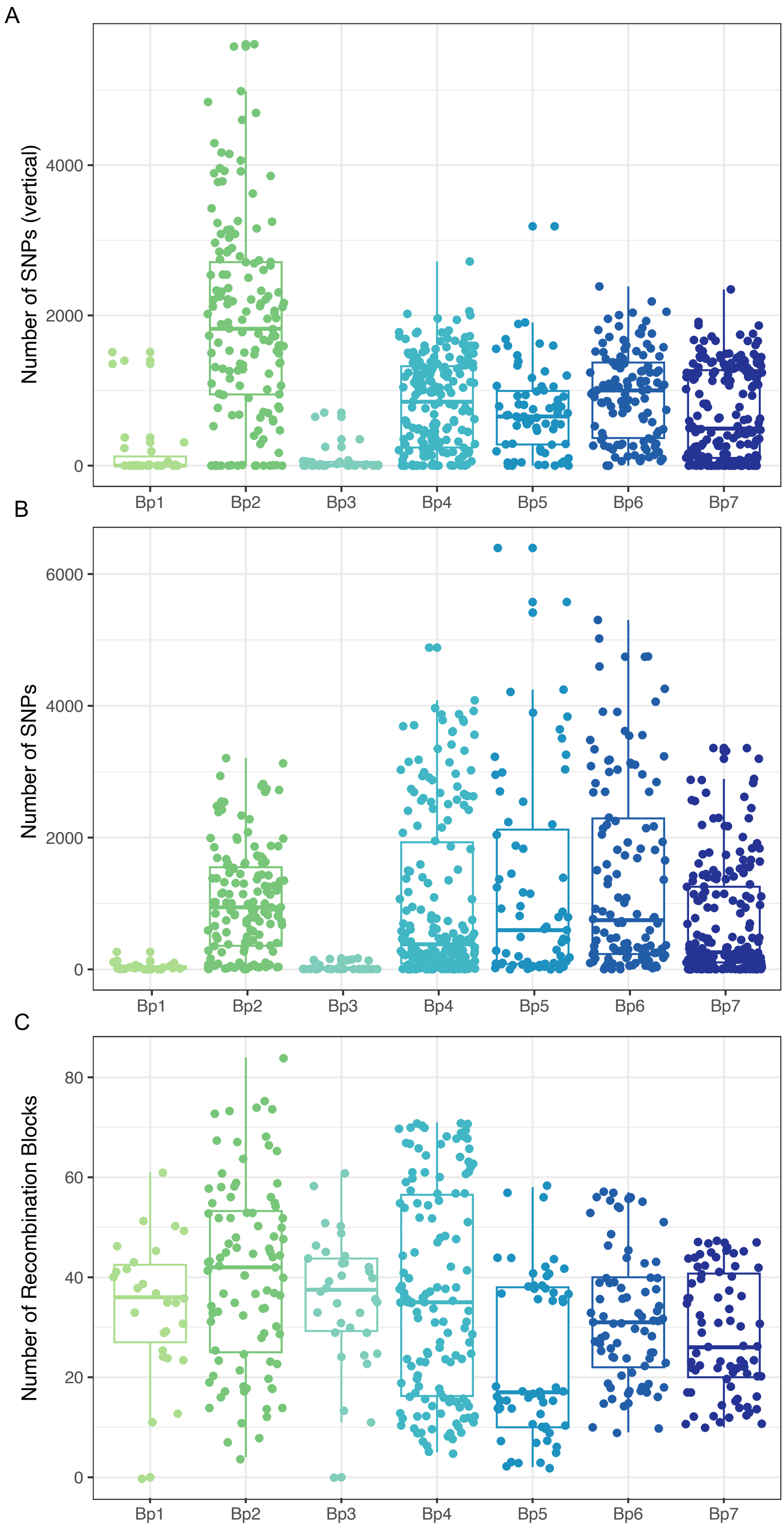

A
Number of SNPs (vertical)
0
2000
4000
Bp1
Bp2
Bp3
Bp4
Bp5
Bp6
Bp7
B
Number of SNPs
0
2000
4000
6000
Bp1
Bp2
Bp3
Bp4
Bp5
Bp6
Bp7
C
Number of Recombination Blocks
0
20
40
60
80
Bp1
Bp2
Bp3
Bp4
Bp5
Bp6
Bp7

Supplementary Figure 3. A-C. Boxplot showing the number of vertical SNPs (A), all SNPs (B), and number of recombination blocks (C) across the different *S. uberis* phylogroups. Each point represents an individual *S. uberis* isolate. The middle line represents the median, and the lower/upper lines represent the 25th and 75th percentiles.

A

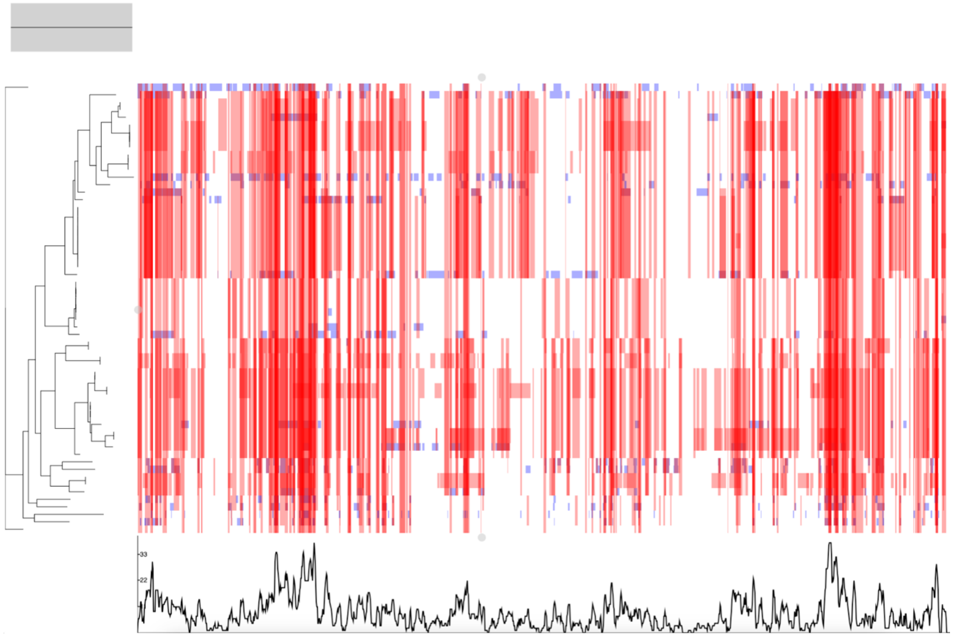
33
22

B

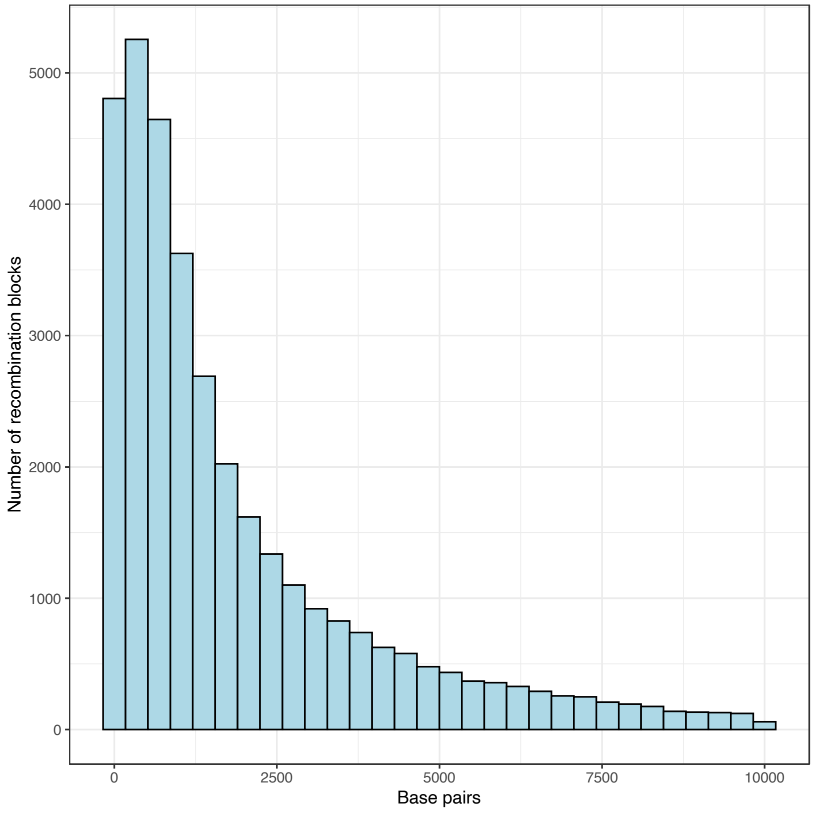
5000
4000
3000
2000
1000
0
Number of recombination blocks
0
2500
5000
7500
10000
Base pairs

Supplementary Figure 4A. Recombination hotspots in the whole-genome alignment. The phylogenetic tree was computed on the recombination sites free alignment for phylogroup 2. The detected recombination hotspots: red blocks represent ancestral recombination while the blue blocks represent specific recombination in one isolate. The black line at the bottom of the figure corresponds to SNP density. **B.** Size distribution of intra-phylogroup recombination lengths. Recombination blocks were defined using a sliding-window approach based on intra-phylogroup mapping.

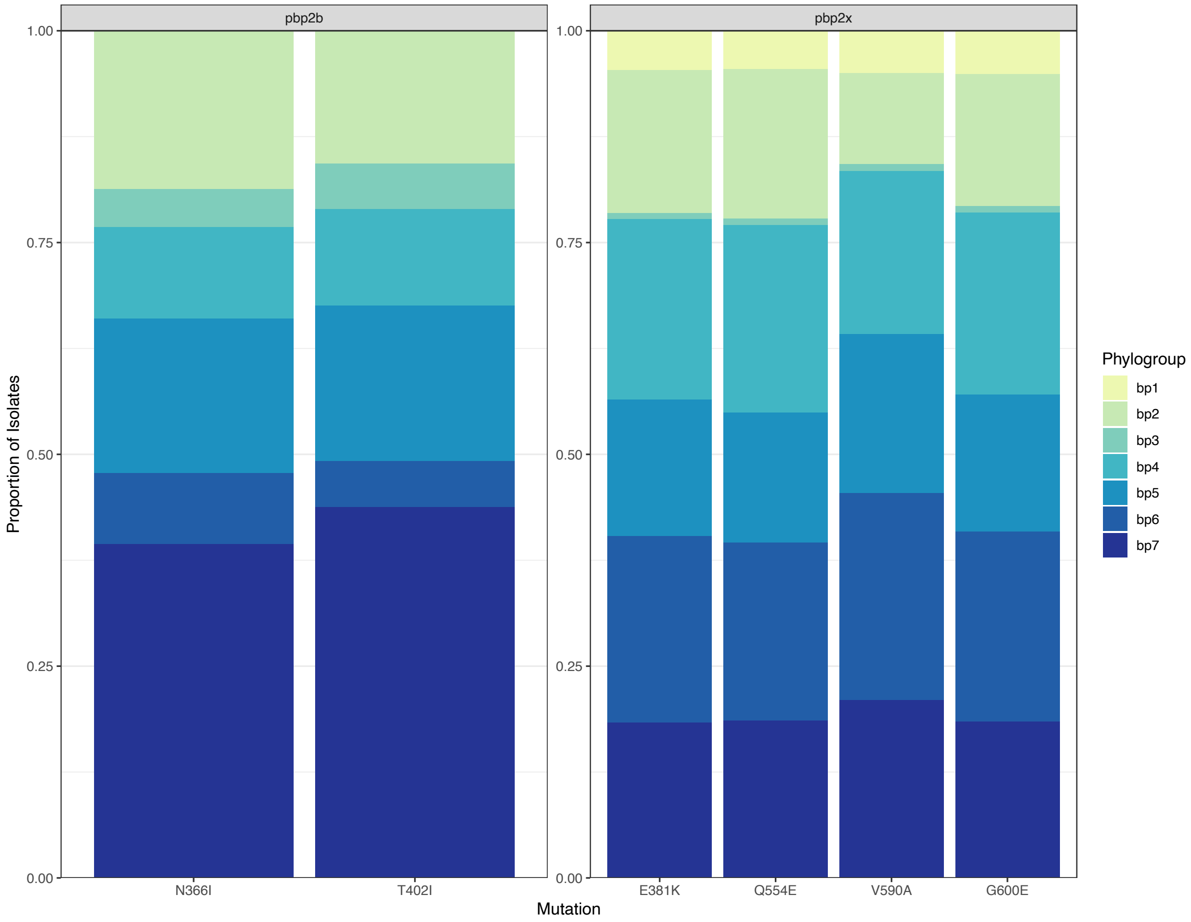

Supplementary Figure 5. Proportion of *pbp* alleles that were present in each phylogroup. No isolates harboured the A227T or G386R mutations in *pbp1a*.

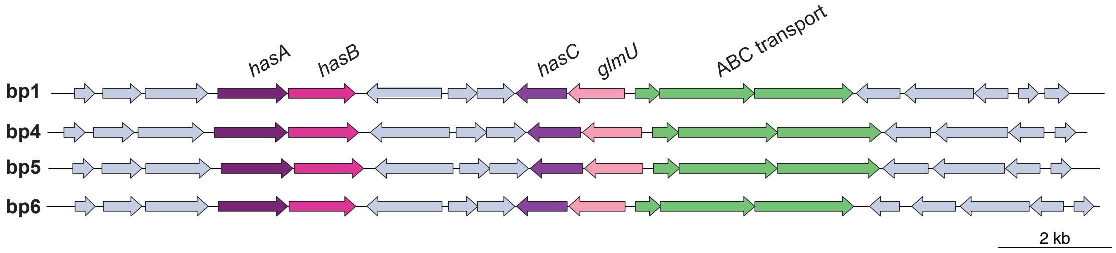

Supplementary Figure 6. Example *hasAB* capsule locus structures for representative *S. uberis* isolates from the major phylogroups. Coding sequences are represented as arrows and coloured by predicted function of the protein product and labelled with gene names relevant to the capsule.
